# The combined supplementation of quercetin and *Ligilactobacillus salivarius* improves production performance, lipid metabolism, and ileal health in late-phase laying hens

**DOI:** 10.1016/j.psj.2025.105286

**Published:** 2025-05-10

**Authors:** Pan Xiao, Qian Li, Leiqing Wang, Guangpeng Sun, Haifeng Hou, Xiaofei Han, Jia Feng, Yuna Min

**Affiliations:** a College of Animal Science and Technology, Northwest A&F University, Yangling 712100, Shaanxi, PR China; b Institute of Animal Husbandry and Veterinary Medicine of Hebei Province, Baoding 071000, Hebei, PR China; c Baoding Vocational and Technical College, Baoding 071051, Hebei, PR China; d Huayu Agricultural Science and Technology Co., Ltd, Handan, 057300, Hebei, PR China

**Keywords:** *Ligilactobacillus salivarius*, quercetin, laying hen, lipid metabolism, ileal health

## Abstract

We studied the effects of a combination of quercetin and *Ligilactobacillus salivarius* (***L. salivarius***) on production performance, lipid metabolism, and ileal health in late-phase laying hens. In a 12-week feeding trial, 480 healthy 50-week-old laying hens received a basal diet supplemented with various treatments: 0 (CON), 0.2 g/kg quercetin and 0.5 × 10^8^ CFU/kg *L. salivarius* (LD), double dose of group LD (MD), triple dose of group LD (HD), and 1.46 g/kg quercetin microcapsules (0.4 g/kg quercetin content matching the group MD) and 1 × 10^8^ CFU/kg *L. salivarius* (group BM). Results showed that the laying rate was significantly higher, feed-to-egg ratio was significantly lower with supplementation (*P* < 0.05), with no changes in egg quality. Supplementation reduced serum TG, TC, HDL-C, and LDL-C levels, along with a significant reduction in the abdominal fat ratio (*P* < 0.05). Compared with the CON group, the HD and BM groups had lower TG and TC levels and reduced expression levels of *ACC-α* and *SREBP1* in the liver (*P* < 0.05). In the ileum, the MD, HD, and BM groups also showed, reduced V/C ratios and histopathological scores (*P* < 0.05), increased intestinal barrier gene expression, including *occludin, claudin-1, Muc2*, and *ZO-1* (*P* < 0.05), and higher expression levels of *IL-10* and *IL-4* (*P* < 0.05), alongside lower expression levels of *IL-6* and *IL-2* (*P* < 0.05). Additionally, dietary supplementation with quercetin and *L. salivarius* increased the abundance of Lactobacillales and *Ligilactobacillus*. Overall, the combination of quercetin and *L. salivarius* improved laying rate while reducing feed-to-egg ratio and abdominal fat ratio, likely mediated by enhanced hepatic lipid metabolism and improved gut immune status. These findings provide novel insights into the combined supplemation for enhancing poultry production efficiency.

## Introduction

Laying hen have a peak egg production period, during they undergoes high-intensity metabolism (Gu et al., 2021). After this process, the production performance in laying hens will be significantly decreased, which is mainly manifested as a decrease in laying rate and an increase in feed-to-egg ratio (Fathi et al., 2019; Schmucker et al., 2021). During the aging process and under the influence of various factors, lipid metabolism disorders result in excessive deposition of liver fat and abdominal fat, which further affect the production performance of laying hens (Li et al., 2015; Shini et al., 2019). Furthermore, the composition of the gut microbiota in laying hens undergoes substantial changes with advancing age (Liu et al., 2021; Wang et al., 2021). During the late laying period, the equilibrium of gut flora can be readily disrupted, leading to an imbalance that serves to weaken the gut barrier function of laying hens, rendering them more predisposed to inflammatory responses, ultimately leading to a decline in production performance (Xiao et al., 2021; Zhuang et al., 2024). Consequently, it is essential to enhance lipid metabolism and intestinal health in laying hens in order to cope with the performance loss in the late laying period.

*Ligilactobacillus salivarius* (***L. salivarius***) is a facultative anaerobic Gram-positive bacterium and is considered to be a symbiotic bacterium in the chicken gut (Lee et al., 2017;Xu et al., 2016). *L. salivarius* has been demonstrated to improve the production performance of laying hens through regulating gut microbiota and lipid metabolism (Liu et al., 2022a; Zhu et al., 2022; Xu et al., 2022). Quercetin can exert anti-inflammatory and energy metabolism regulatory effects by directly interacting with various intracellular proteins (e.g., xanthine oxidase, phosphoinositide 3-kinase, transthyretin) (Nair et al., 2006; Choi et al., 2008; Eid et al., 2015). Additionally, quercetin modulates host metabolism by regulating the gut microbiota (Feng et al., 2023a; Zhu et al., 2024). Previous studies have reported that quercetin supplementation (200-600 mg/kg) in laying hens improves performance by delaying inflammatory responses and regulating gut microbiota (Feng et al., 2023a; Liu et al., 2023a). Based on the complementary and superimposed effects of quercetin and *L. salivarius* on metabolic, immune, and microbial regulatory pathways, their combined application represents a highly promising strategy. As previously noted, the combination of plant extracts and probiotics exhibits a more pronounced outcome in laying hens compared to their individual applications (Wang et al., 2022; Obianwuna et al., 2023).

Nevertheless, the efficacy and application of quercetin are always limited due to its poor solubility, structural instability, and extensive degradation loss in the stomach (Wang et al., 2016; Lou et al., 2023). Thus, there are increasing interests in encapsulating quercetin in various carriers for intestine targeted delivery, in which the compounds can be protected in the stomach and can be released in the intestine at a controlled rate (Kandemir et al., 2022). Previous studies have demonstrated that encapsulation not only enhances quercetin's bioavailability but also potentiates its synergistic interaction with hindgut microbes (Liu *et al.*, 2023b).

This study will evaluate the effects of dietary supplementation of quercetin combined with *L. salivarius* on production performance, lipid metabolism, ileal health of laying hens during the late laying period, with the objectives of determining the optimal supplementation dosage and further exploring how microencapsulation enhances the efficacy of quercetin's application.

## Material and methods

### Quercetin determination

Quercetin was obtained from a commercial supply (purity≥95%, Product number: Q111274, CAS No. 117-39-5, Moligand™, Shanghai, China). According to previous reported method and with appropriate modifications (Simonetti et al., 2022), a high-performance liquid chromatography (**HPLC**) system (1260 Infinity III, Agilent, CA, USA) was used to quantify quercetin. In short, inject each sample (5 μL) into an HPLC column (ZORBAX SB-C118, 2.1 × 150 mm, 5 µm) with a column temperature of 30°C. Elution was performed in gradient mode, using the mobile phase MeOH-H_3_PO_4_ 10^−3^ mL/L and detection wavelength is 370 nm.

### Strain and growth conditions

*L. salivarius* CRS7-1 was donated by Academy of National Food and Strategic Reserves Administration, which have been isolated from ileum of laying hens. Bacteria were grown on De Man-Rogosa-Sharpe (MRS) medium in anaerobic conditions (anaerobic conditions were achieved by the use of anaerobic jars with 5% CO_2_) at 37°C.

### In vitro interaction evaluation

Prepare MRS medium with quercetin concentrations of 0 mg/mL, 0.2 mg/mL, 0.4 mg/mL, and 0.6 mg/mL, respectively. For the evaluation of the in vitro interaction between quercetin and *L. salivarius*, we referred the co-culture method described by previous studies with minor modifications to the co-culture time (Dos Santos et al., 2019; Bijle et al., 2020). Inoculate equal amounts (1% inoculum) of *L. salivarius* in four different culture media, and cultivate at 37°C. Bacterial solution was collected at the appropriate time points and count the colony-forming unit (CFU). CFU counts were performed using the spread plate method as previous reported (BNO Team, 2024). In briefly, 30 μL of each diluted sample (prepared in sterile PBS) was evenly spread onto pre-warmed MRS agar plates using a sterile glass spreader, plates were incubated under anaerobic conditions at 37°Cfor 24 hours. Only plates with 30–300 colonies were considered valid to ensure statistical reliability.

### In vitro digestion of quercetin

Quercetin microcapsules entrusted to King Techina Biotechnology Co., Ltd. for embedding production. The embedding process used fluidized bed technology, and the embedding wall materials selected were sodium carboxymethyl cellulose, acrylic resin, carboxymethyl cellulose, etc. Sodium carboxymethyl cellulose (SCMC) was chosen due to its adhesive and emulsifying properties, while acrylic resin has pH-responsive release and stomach resistance (Mukhopadhyay et al., 2014; Gadalla et al., 2016). According to the manufacturer's instructions, the encapsulation rate is greater than 90%. The size of the encapsulated quercetin microcapsules ranged from 250 mm to 425 mm. The content of quercetin was 27.5%, the retention rate of simulated gastric fluid (**SGF**) was 86.9% after 4 h, and the release rate of simulated intestinal fluid (**SIF**) was 92.8% after 12 h.

To evaluate the process of quercetin microcapsules during chicken gastrointestinal digestion, SGF and SIF were prepared following the procedure outlined (Kim et al., 2021), with slight adjustments. SGF was prepared with mixed solution of 16.9 mmol/L NaCl, 9.6 mmol/L KCl, 1550 U/mL gastric protease and 10 mmol/L HCl, followed by pH adjustment to 2.0 by adding HCl at 41°C. 20 mg quercetin and 73 mg quercetin microcapsules were added to 50 mL SGF, respectively, with temperature of 41°C and a speed of 80 rpm to SGF. Solution was collected (1 mL) from SGF at the appropriate time points and replenished with respective SGF. After 60 min of SGF digestion, 4.29 mmol NaCl, 0.4675 mmol KCl, 8.5 mmol NaH_2_PO_4_, and 1.5 mmol Na_2_HPO_4_ were added to the solution. The pH was adjusted to 6.3-6.7 using 1 mol/L Na_2_CO_3_ solution, and 10 mg/mL trypsin was added to solution to simulated intestinal digestion. Solution was collected (1 mL) from SIF at the appropriate time points and replenished with respective SIF. The digestive solution was diluted tenfold with methanol, centrifuged at 7, 000 rpm for 10 min, and the supernatant was filtered through a 0.22 μm filter. The quercetin content was determined using HPLC as previously described.

### Feed preparation

The strain was processe d into freeze-dried bacterial powder using established methods in our laboratory, with the viable count verified to be 1 × 10^11^ CFU/g. To ensure the sufficient viability of *L. salivarius* CRS7-1, the experimental diet was prepared weekly. Existing literature indicates that the supplementation range for quercetin is 200–600 mg/kg, while the common dosage for *L. salivarius* is 1 × 10^8^ CFU/kg (Liu et al., 2014; Zhu et al., 2022; Xu et al., 2022; Liu et al., 2024; Wei et al., 2024). Based on these findings, we designed three gradient dosages for the combined supplementation of quercetin and *L. salivarius*. We calculated the precise quantities of *L. salivarius*, quercetin, and basal diet required for formulating the experimental diet. The additives were then diluted with a portion of the basal diet and thoroughly mixed with the remaining feed.

### Animal experimental design

The trial was conducted in accordance with the National Research Council Guide for the Care and Use of Laboratory Animals, and the regulations of the Ani-mal Ethical and Welfare Committee of Northwest A&F University (Approval No. DK2022061). A total of 480 healthy 48-week-old Hy-Line variety brown laying hens were randomly assigned to 5 groups with 8 replicates of 12 chickens each. Production performance prior to the experiment were similar across all the replicates. In an environmentally controlled chicken coop, chickens can freely drink water and mash feed in a 3-tier cage (45 cm × 45 cm × 45cm; 3 chickens per cage) with controlled ventilation and lighting (16L: 8D). Chickens in the control group (**CON**) was fed with a basal diet formulated according to NRC (1994) recommendations and Chinese Feeding Standard of Chicken (Table 1). The experimental group was fed with basal diet supplemented with 0.2 g/kg quercetin and 0.5 × 10^8^ CFU/kg *L. salivarius* (**LD**), 0.4 g/kg quercetin and 1 × 10^8^ CFU/kg *L. salivarius* (**MD**), 0.6 g/kg quercetin and 1.5 × 10^8^ CFU/kg *L. salivarius* (**HD**), and 1.46 g/kg quercetin microcapsules and 1 × 10^8^ CFU/kg *L. salivarius* (**BM**). After 2 weeks of preliminary testing, a formal period was 12 weeks.

**Table 1:** Ingredients and nutrient levels of basic diets (air-dried basis).

Ingredients	Contents (%)	Nutrient levels^2^	
Corn	64.15	Metabolizable energy (MJ/kg)	11.20
Soybean meal	22.70	Crude protein	15.80
Limestone	7.50	Calcium	3.78
Soybean oil	0.65	Available phosphorus	0.37
NaCl	0.15	Lysine	0.82
Choline	0.13	Methionine	0.40
DL-Methionine	0.12	Methionine+cystine	0.64
Premix^1^	4.60		
Total	100.00		

1 Premix provided the following per kg of the diet: vitamin A, 12,500 IU; vitamin D3, 4125 IU; vitamin E, 15 IU; vitamin K3, 2 mg; thiamine, 1 mg; riboflavin, 8.5 mg; calcium pantothenate, 11 mg; niacin, 32.5 mg; pyridoxine; 8 mg; biotin, 0.5 mg; folic acid, 1.25 mg; vitamin B12, 0.02 mg; Mn (MnSO_4_), 65 mg; I (KI), 1 mg; Fe (FeSO_4_), 60 mg; Cu (CuSO_4_), 8 mg; Zn (ZnO), 66 mg; phytase, 500 mg.

2 The nutrient levels are calculated values.

### Performance measurements

Record daily egg production, egg weight, feed consumption, and mortality rate up to 61 weeks of age. Based on these data, egg weight (g), laying rate (%), egg mass, average daily feed intake (**ADFI**), and feed-to-egg ratio (total feed intake divided by total egg mass) were calculated.

### Sample collection

On the last day of the experiment, 8 birds were randomly selected from each group (1 from each replicate) for sampling. Blood samples were collected from the brachial vein after a 12-h fast and then placed it at 37°C to precipitate serum. The hens selected for blood sample collection were weighed and euthanized by cervical dislocation. The livers, spleens and abdominal fat were isolated and weighed; then, their weights relative to body weight was measured, and data were expressed as a percentage of body weight. Segments from the mid-ileum were collected and fixed in 10% neutral formalin for histological analysis. The contents of the ileum were collected in cryopreservation tubes, immediately placed in liquid nitrogen for short-term storage, and subsequently transferred to -80°C for freezing prior to DNA extraction.

### Egg quality

Egg quality testing was conducted on a random selection of five eggs from each replicate every four weeks (at 53, 57, and 61 weeks of age). All the eggs from the final measurement week were collected, and the following parameters were assessed: egg weight, eggshell strength, eggshell thickness, Haugh unit, and yolk color, using the Egg Tester Ultimate™ (Orka Food Technology, Israel).

### Serum biochemical analysis

The serum from each treatment group (n = 8) was collected. The levels of alanine aminotransferase (**ALT**), aspartate aminotransferase (**AST**), free fatty acid (**FFA**), triglycerides (**TG**), total cholesterol (**TC**), high-density lipoprotein cholesterol (**HDL-C**), and low-density lipoprotein cholesterol (**LDL-C**) were measured using a chemistry analyzer (Indiko, Thermo Fisher Scientific, Waltham, MA, USA). FFA assay kit (Beyotime Biotechnology, Shanghai, China) was used to determine the FFA concentration following the kit instructions. The remaining biochemical indicators were analyzed using commercial kits instructions of the relevant kits (Nanjing Jiancheng Biological Engineering Institute, China).

### Gut histomorphology analysis

Fixed jejunum and ileum specimens were dehydrated, embedded in paraffin, sectioned into 5 μm slices, and subsequently stained with hematoxylin and eosin **(H&E**). To evaluate villus height and crypt depth, 15 oriented villi and their corresponding crypts were selected for analysis.

### DNA extraction and 16S rRNA sequencing

Microbial genomic DNA was extracted from the samples using the Stool DNA Kit (200) (D4015-02, Omega Bio-Tek, Norcross, GA, USA) according to the manufacturer's instructions. The bacterial 16S rRNA V3-V4 region was amplified using qualified DNA as the template, along with the primers 341F (5′-CCTACGGGNGGCWGCAG-3′) and 805R (5′-GACTACHVGGGTATCTAATCC-3′) through polymerase chain reaction (**PCR**). The amplified products were purified using AMPure XT beads (Beckman Coulter Genomics, Danvers, Massachusetts, USA) and quantified with a Qubit fluorometer (Invitrogen, Carlsbad, CA, USA). Sequencing was performed on a NovaSeq 6000 platform (Illumina, San Diego, CA, USA) following standard protocols. The raw paired-end reads were quality-filtered using Trimmomatic software. Subsequently, DADA2 (https://benjjneb.github.io/dada2/) was employed to denoise the data. An OTU-like table was constructed after clustering, utilizing the concept of amplicon sequence variants (**ASVs**) to obtain the final ASVs feature sequences. Data analysis was conducted using the OmicStudio cloud platform (LC Bio Technologies, Hangzhou, China).

### RNA extraction and quantitative PCR

RNA extraction and qPCR manipulation methods were consistent with those previously reported (Feng et al., 2023a)**.** Briefly, RNA was extracted using the TRIzol method, reverse-transcribed into cDNA, and the mRNA expression levels were quantified using the SYBR Green I dye method. Each sample was analyzed in triplicate, and the relative mRNA expression levels were calculated using β-actin as an internal control by the 2^−ΔΔCT^ method. All primers were designed using the NCBI database, and primer specificity and amplification efficiency were determined by Primer-BLAST and melt curves. The primer sequences used in this study are shown in Table 2.

**Table 2:** Oligonucleotide primers used for gene expression by quantitative real time PCR.

Genes	Primer sequence (5′-3′)	Accession No.	Tm (°C)	Product length(bp)
*IL-2*	F: CCAACTGAGACCCAGGAGTG	NM_204153.2	60	174
	R: ACTTCCGGTGTGATTTAGACCC		60	
*IL-6*	F: TCGCCTTTCAGACCTACCTG	NM_204628.2	59	180
	R: TCAGATTGGCGAGGAGGGA		60	
*IL-10*	F: ATGCTGCGCTTCTACACAGA	NM_001004414.4	60	205
	R: TCCCGTTCTCATCCATCTTCTC		59	
*IL-4*	F: GGAGAGGTTTCCTGCGTCAA	NM_001007079.2	60	200
	R: GTGGGACATGGTGCCTTGAG		61	
*TNF-α*	F: GAGCGTTGACTTGGCTGTC	XM_046927265.1	59	64
	R: AAGCAACAACCAGCTATGCAC		60	
*FXR*	F: TGTGAAGGATGCAAAGGGTTCT	NM_204113.3	60	145
	R: TGCCCATTTGCTTGCATTTCC		61	
*PPAR-α*	F: AAGATGGGATGCTGGTAGCC	NM_001001464.1	60	192
	R: GACCAGGACGATCTCCACAG		60	
*SREBP1*	F: GATCATGCGGCGACCGA	XM_046927252.1	60	289
	R: CGAACAGCCCTGAGAAGTCA		60	
*FASN*	F: TGGCATACGAACTGGCTACC	NM_205155.4	60	244
	R: GGTGCCTGAATACTTGGGCT		60	
*LXR-α*	F: TCGGCGCTACAATCCAGAGA	XM_046917664.1	61	209
	R: GCACATTCGGTCGGTCTGC		62	
*ZO-1*	F: GGAGATTCCGAGGTTTGCGT	XM_046925214.1	60	129
	R: TGAGAAACCCAGCTTCCCGA		61	
*Claudin-1*	F: GATCCAGTGCAAGGTGTACGA	NM_001013611.2	60	181
	R: AAAGACAGCCATCCGCATCT		60	
*Claudin-2*	F: ATCTCCAGCCATCTCTGTAACCT	NM_001277622.1	61	151
	R: CTCTCCCGCACGTTTACCTTT		61	
*Occludin*	F: GTCTGTGGGTTCCTCATCGT	XM_046904539.1	59	156
	R: GTTCTTCACCCACTCCTCCA		59	
*Muc2*	F: AGATGCACTGATGGTGGAGC	XM_040701656.2	60	249
	R: GCAGAGGAGGTCAGCAACTT		60	
*β-actin*	F: ATTGTCCACCGCAAATGCTTC	NM_205518.2	60	113
	R: AAATAAAGCCATGCCAATCTCGTC		60	

Abbreviations: *FXR*, farnesoid X receptor; *PPAR-α*, peroxisome proliferator activated receptor alpha; *SREBP1*, sterol-regulatory element-binding protein 1; *FASN*, fatty acid synthase; *LXR-α*, liver X receptors alpha; *ACC-α*, acetyl-CoA carboxylase alpha.

### Statistical analysis

Data were presented as mean ± SEM. The data were analyzed using one-way analysis of variance (**ANOVA**), followed by Duncan’s Multiple Range Test, utilizing SPSS 27 (SPSS Inc., Chicago, IL). P values less than 0.05 were considered statistically significant.

## Results

### Evaluation of the interaction between quercetin and L. salivarius

**Fig. 1:**
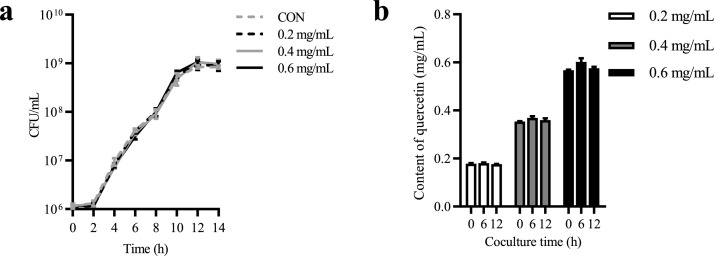
The interaction between quercetin and *L. salivarius*. Growth curve of *L. salivarius* in MRS medium with varying concentrations of quercetin (a). The quercetin content during the growth of *L. salivarius* (b). Values are presented as mean ± SEM (n = 3).

The growth curves of *L. salivarius* in varying concentrations of quercetin did not exhibit significant changes, indicating that quercetin cannot affect the in vitro growth performance of *L. salivarius* (Fig. 1a). Furthermore, there was no notable alteration in the quercetin content during mixed culture (Fig. 1b), indicating the absence of interaction between quercetin and *L. salivarius* in vitro.

#### Quercetin gastrointestinal release and degradation

**Fig. 2:**
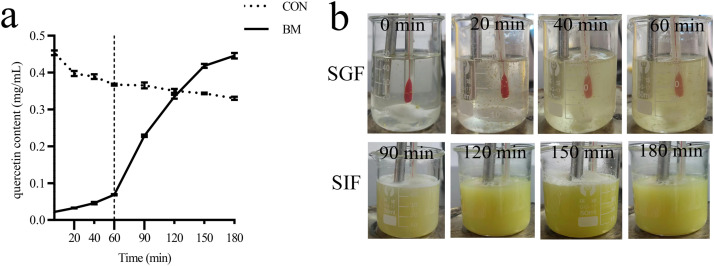
Dissolution and release of quercetin in simulated gastric fluid (SGF) and simulated intestinal fluid (SIF). Quercetin content in SGF and SIF (a). Dissolution and release of quercetin microcapsules in SGF and SIF (b). CON, quercetin; BM, quercetin microcapsules. Values are presented as mean ± SEM (n = 3).

The changes in quercetin content in SGF and SIF are illustrated in Fig. 2. The quercetin content significantly decreased by 19.07% in SGF within 60 mis (*P* < 0.05). The encapsulated group released only 10% of quercetin during the SGF phase, while a rapid release of quercetin occurred upon entering the SIF (Fig. 2a). Fig. 2b demonstrates the dissolution behavior of microencapsulated quercetin in both SGF and SIF. When these microcapsules were placed in SGF, the dissolution was very slow, and the quercetin microcapsules remained intact after 60 min. In contrast, a more pronounced time-dependent dissolution of quercetin microcapsules and subsequent quercetin release was observed in SIF. This indicates that quercetin, when subjected to encapsulation, can effectively prevent degradation in the stomach while facilitating rapid release in the intestine.

#### Production performance and egg quality

As shown in Table 3, the MD, HD, and BM groups exhibited a significant improvement in laying rates compared to the CON group from week 51 to week 61 (*P* < 0.05). Furthermore, the LD, MD, HD, and BM groups demonstrated a substantial reduction in ADFI and feed-to-egg ratio relative to the CON group from week 51 to week 61 (*P* < 0.05). The results of the egg quality tests are presented in Table 4, and no significant differences were observed among all groups.

**Table 3:** Effect of quercetin and *L. salivarius* supplementation on production performance of the late-phase of laying hens^1^.

Items	Groups^2^					SEM	*P*-value
	CON	LD	MD	HD	BM		
50 to 53 W							
Laying rate (%)	87.50	89.78	90.38	88.99	90.28	1.365	0.218
ADFI (g/hen/day)	134.92	133.45	134.63	133.53	135.90	1.929	0.693
Average egg weight (g)	65.19	65.48	65.46	65.35	65.05	0.185	0.101
Egg mass (g/hen/day)	65.09	65.18	65.59	65.66	64.85	0.702	0.756
Feed-to-egg ratio (kg:kg)	2.38	2.29	2.28	2.30	2.33	0.050	0.266
54 to 57 W							
Laying rate (%)	89.09	89.88	91.12	90.97	91.77	0.999	0.079
ADFI (g/hen/day)	128.94^a^	128.37^a^	126.62^b^	125.75^c^	126.13^bc^	0.370	<0.001
Average egg weight (g)	65.80	65.66	65.99	66.11	65.80	0.218	0.259
Egg mass (g/hen/day)	65.99	65.83	66.59	66.11	65.55	0.637	0.582
Feed-to-egg ratio (kg:kg)	2.20^a^	2.17^a^	2.10^bc^	2.09^c^	2.10^c^	0.032	0.006
58 to 61 W							
Laying rate (%)	83.10^b^	86.31^a^	87.59^a^	88.62^a^	89.23^a^	1.476	0.002
ADFI (g/hen/day)	120.05^a^	119.20^ab^	118.78^b^	116.65^c^	117.43^c^	0.518	<0.001
Average egg weight (g)	65.16	65.61	65.31	65.62	65.41	0.220	0.183
Egg mass (g/hen/day)	65.16	65.54	65.34	65.61	65.08	0.638	0.903
Feed-to-egg ratio (kg:kg)	2.23^a^	2.13^a^	2.09^bc^	2.02^c^	2.03^C^	0.043	<0.001
50 to 61 W							
Laying rate (%)	86.35^b^	88.43^a^	89.49^a^	89.44^a^	90.31^a^	0.966	0.003
ADFI (g/hen/day)	129.02^a^	128.06^b^	127.71abc	126.25^c^	127.40^bc^	0.746	0.014
Average egg weight (g)	65.37	65.57	65.58	65.66	65.38	0.120	0.056
Egg mass (g/hen/day)	65.38	65.52	65.80	65.78	65.16	0.577	0.778
Feed-to-egg ratio (kg:kg)	2.26^a^	2.19^b^	2.15^bc^	2.12^c^	2.14^bc^	0.029	<0.001

1 Each mean represents 8 replicates.

2 CON, hens received basal diet; LD, hens received basal diet supplemented with 0.2 g/kg quercetin and 0.5 × 10^8^ CFU/kg *L. salivarius*; MD, hens received basal diet supplemented with 0.4 g/kg quercetin and 1 × 10^8^ CFU/kg *L. salivarius*; HD, hens received basal diet supplemented with 0.6 g/kg quercetin and 1.5 × 10^8^ CFU/kg *L. salivarius*; BM, hens received basal diet supplemented with 1.46 g/kg quercetin microcapsules (equivalent to 0.4 g/kg quercetin) and 1 × 10^8^ CFU/kg *L. salivarius*.

a-c Values within a row with no common superscripts differ significantly (*P* < 0.05). Feed-to-egg ratio: feed-to-egg ratio was calculated as total feed intake divided by total egg mass (kg/kg). Abbreviations: SEM, standard error of mean; ADFI, average daily feed intake.

**Table 4:** Effect of quercetin and L. salivarius on egg quality of the late-phase laying hens^1^.

Items	Groups^2^					SEM	*P*-value
	CON	LD	MD	HD	BM		
53 W							
Egg weight (g)	65.95	65.93	66.61	66.90	66.52	1.427	0.947
Eggshell strength (kg)	4.44	5.01	4.71	4.82	5.01	0.300	0.307
Albumen height (mm)	7.09	7.40	7.43	7.853	7.84	0.339	0.157
Haugh unit	81.41	83.65	86.04	86.24	83.09	2.384	0.230
Yolk color	6.50	6.31	6.38	6.19	6.28	0.200	0.616
Eggshell thickness (mm)	0.39	0.39	0.38	0.39	0.38	0.007	0.220
Egg shape index	0.77	0.77	0.77	0.77	0.78	0.007	0.380
57 W							
Egg weight (g)	65.52	65.08	63.86	64.59	65.72	1.758	0.833
Eggshell strength (kg)	4.57	4.228	4.11	4.50	4.29	0.525	0.898
Albumen height (mm)	9.80	9.74	9.52	9.42	9.89	0.380	0.711
Haugh unit	97.55	96.86	98.64	97.08	97.43	1.707	0.859
Yolk color	7.56	7.25	7.31	7.31	7.56	0.320	0.778
Eggshell thickness (mm)	0.41	0.39	0.39	0.39	0.40	0.007	0.055
Egg shape index	0.76	0.77	0.77	0.76	0.76	0.010	0.831
61 W							
Egg weight (g)	64.78	65.82	64.52	65.49	66.29	1.095	0.479
Eggshell strength (kg)	4.72	4.57	4.59	4.78	4.60	0.260	0.906
Albumen height (mm)	9.90	9.95	9.89	9.52	9.37	0.241	0.073
Haugh unit	97.63	98.72	97.83	96.71	95.28	1.216	0.079
Yolk color	7.33	6.96	7.13	7.25	7.38	0.263	0.516
Eggshell thickness (mm)	0.39	0.39	0.38	0.37	0.39	0.006	0.083
Egg shape index	0.75	0.76	0.76	0.75	0.76	0.007	0.294

1 Each mean represents 8 replicates.

2 CON, hens received basal diet; LD, hens received basal diet supplemented with 0.2 g/kg quercetin and 0.5 × 10^8^ CFU/kg *L. salivarius*; MD, hens received basal diet supplemented with 0.4 g/kg quercetin and 1 × 10^8^ CFU/kg *L. salivarius*; HD, hens received basal diet supplemented with 0.6 g/kg quercetin and 1.5 × 10^8^ CFU/kg *L. salivarius*; BM, hens received basal diet supplemented with 1.46 g/kg quercetin microcapsules (equivalent to 0.4 g/kg quercetin) and 1 × 10^8^ CFU/kg *L. salivarius*. Abbreviations: SEM, standard error of mean.

#### Organ index

As illustrated in Table 5, the MD, HD, and BM groups demonstrated a significant reduction in the abdominal fat ratio of laying hens compared to the CON group (*P* < 0.01). Furthermore, no significant alterations were observed in the liver and spleen indices.

**Table 5:** Effect of dietary quercetin and L. salivarius supplementation on tissue index of the late-phase laying hens^1^.

Items	Groups^2^					SEM	*P*-value
	CON	LD	MD	HD	BM		
Liver index (%)	2.50	2.43	2.41	2.47	2.42	0.114	0.909
Spleen index (%)	0.14	0.13	0.13	0.12	0.12	0.013	0.523
Abdominal fat (%)	2.94^a^	2.79^a^	2.27^b^	2.28^b^	2.20^b^	0.215	0.003

1 Each mean represents 8 replicates.

2 CON, hens received basal diet; LD, hens received basal diet supplemented with 0.2 g/kg quercetin and 0.5 × 10^8^ CFU/kg *L. salivarius*; MD, hens received basal diet supplemented with 0.4 g/kg quercetin and 1 × 10^8^ CFU/kg *L. salivarius*; HD, hens received basal diet supplemented with 0.6 g/kg quercetin and 1.5 × 10^8^ CFU/kg *L. salivarius*; BM, hens received basal diet supplemented with 1.46 g/kg quercetin microcapsules (equivalent to 0.4 g/kg quercetin) and 1 × 10^8^ CFU/kg *L. salivarius*.

a-b Values within a row with no common superscripts differ significantly (*P* < 0.05). Abbreviations: SEM, standard error of mean.

#### Serum biochemical parameters

As shown in Table 6, the serum levels of LDL-C (*P* = 0.053), TG (*P* = 0.004), and TC (*P* = 0.051) were lower in the MD, HD, and BM groups. Additionally, the serum enzyme activity of AST in the BM group also decreased significantly (*P* = 0.0 11). Compared to the CON group, the serum enzyme activity of ALT in the supplementation group decreased significantly (*P* < 0.001).

**Table 6:** Effect of dietary quercetin and L. salivarius supplementation on serum biochemical indices of the late-phase laying hens 1.

Items3	Groups^2^					SEM	*P*-value
	CON	LD	MD	HD	BM		
HDL-C (mmol/L)	1.39	1.27	0.86	0.68	0.79	0.411	0.137
LDL-C (mmol/L)	1.67	1.63	1.33	1.14	1.02	0.262	0.053
TG (mmol/L)	9.49^a^	6.36^b^	6.34^b^	6.29^b^	5.18^b^	1.063	0.004
TC (mmol/L)	2.11	2.19	1.57	1.68	1.61	0.262	0.051
AST (U/L)	73.54^a^	69.01^a^	65.83^a^	65.57^a^	49.96^b^	6.414	0.011
ALT (U/L)	19.24^a^	8.91^b^	8.66^b^	7.16^b^	7.83^b^	1.931	<0.001

1 Each mean represents 8 replicates.

2 CON, hens received basal diet; LD, hens received basal diet supplemented with 0.2 g/kg quercetin and 0.5 × 10^8^ CFU/kg *L. salivarius*; MD, hens received basal diet supplemented with 0.4 g/kg quercetin and 1 × 10^8^ CFU/kg *L. salivarius*; HD, hens received basal diet supplemented with 0.6 g/kg quercetin and 1.5 × 10^8^ CFU/kg *L. salivarius*; BM, hens received basal diet supplemented with 1.46 g/kg quercetin microcapsules (equivalent to 0.4 g/kg quercetin) and 1 × 10^8^ CFU/kg *L. salivarius*.

a-b Values within a row with no common superscripts differ significantly (*P* < 0.05). Abbreviations: SEM, standard error of mean; HDL-C, high density lipoprotein cholesterol; LDL-C, low density lipoprotein cholesterol; TG, triglyceride; TC, total cholesterol; AST, aspartate aminotransferase; ALT, alanine aminotransferase.

#### Liver lipid parameters and mRNA expression of genes related to liver lipid metabolism

**Fig. 3:**
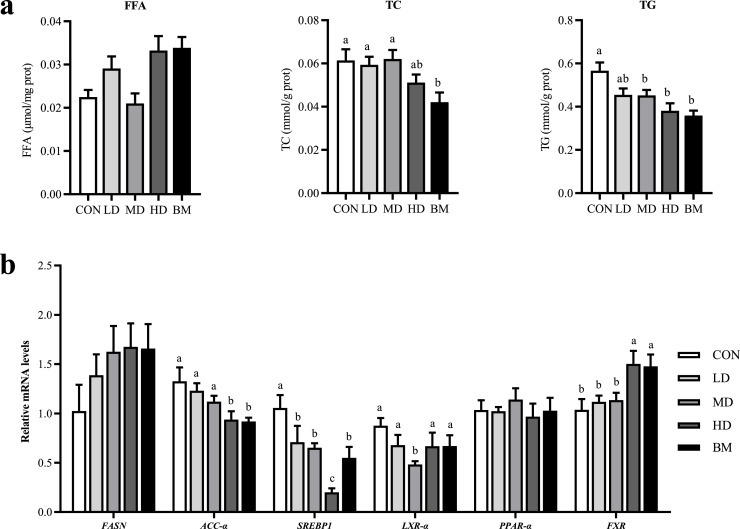
Effects of quercetin and L. salivarius on liver lipid parameters. CON, hens received basal diet; LD, hens received basal diet supplemented with 0.2 g/kg quercetin and 0.5 × 10^8^ CFU/kg *L. salivarius*; MD, hens received basal diet supplemented with 0.4 g/kg quercetin and 1 × 10^8^ CFU/kg *L. salivarius*; HD, hens received basal diet supplemented with 0.6 g/kg quercetin and 1.5 × 10^8^ CFU/kg *L. salivarius*; BM, hens received basal diet supplemented with 1.46 g/kg quercetin microcapsules (equivalent to 0.4 g/kg quercetin) and 1 × 10^8^ CFU/kg *L. salivarius*. ^a-c^ Means with different lowercase superscripts represent significant difference (*P* < 0.05).

As illustrated in Fig. 3, the liver TG and TC contents were significantly lower in the BM and HD groups compared to the CON group (*P* < 0.05). No significant differences in FFA levels were observed among the groups (Fig. 3a). The expression levels of genes associated with hepatic lipid metabolism were subsequently analyzed (Fig. 3b). In comparison to the CON group, the expression levels of the *SREBP1* were significantly reduced in the LD, MD, HD, and BM groups (*P* < 0.05). Additionally, in the HD and BM groups, the expression levels of the acetyl-CoA carboxylase-αlpha (**ACC-α**) gene were significantly decreased, while the expression levels of the farnesoid X receptor (**FXR**) gene were significantly increased relative to the CON group (*P* < 0.05). Meanwhile, the MD group exhibited a notable decrease in the expression level of the liver X receptor-alpha (**LXR-α**) gene (*P* < 0.05).

#### Intestinal morphology

**Fig. 4:**
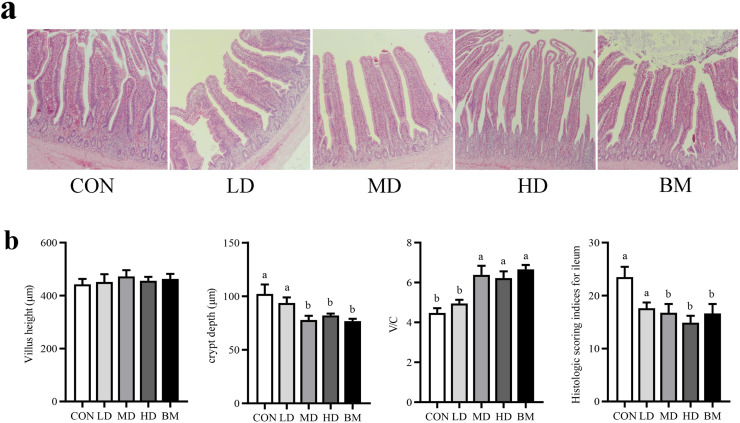
Effects of dietary quercetin and L. salivarius supplementation on gut morphology. Representative photomicrographs of ileum with HE staining (a). Villus length, crypt depth, villus length/crypt depth ratio and histology score of ileum (b). CON, hens received basal diet; LD, hens received basal diet supplemented with 0.2 g/kg quercetin and 0.5 × 10^8^ CFU/kg *L. salivarius*; MD, hens received basal diet supplemented with 0.4 g/kg quercetin and 1 × 10^8^ CFU/kg *L. salivarius*; HD, hens received basal diet supplemented with 0.6 g/kg quercetin and 1.5 × 10^8^ CFU/kg *L. salivarius*; BM, hens received basal diet supplemented with 1.46 g/kg quercetin microcapsules (equivalent to 0.4 g/kg quercetin) and 1 × 10^8^ CFU/kg *L. salivarius*. ^a-c^ Means with different lowercase superscripts represent significant difference (*P* < 0.05).

As shown in Fig. 4, the intestinal mucosa maintained structural integrity across all experimental groups, characterized by well-defined villi and the absence of edema or congestion (Fig. 4a). The length of the villi remained relatively unchanged; however, the depth of the crypts in the ileum was significantly reduced in the MD and BM groups compared to the CON group (*P* < 0.05). Additionally, the villus-crypt ratio was significantly elevated (Fig. 4b). Notably, there was a significant difference in ileal pathology scores, with the HD and BM groups demonstrating marked improvement in intestinal bleeding compared to the CON group (Table 7, Fig. 4b).

**Table 7:** Histologic scoring indices for ileum^1^.

Items	Groups^2^					SEM	*P*-value
	CON	LD	MD	HD	BM		
Lamina propria thickness (μm)	3.75	2.50	2.75	1.75	2.63	0.689	0.094
Epithelial thickness (μm)	1.88	1.50	1.63	1.13	1.29	0.406	0.431
Epithelial cell proliferation	2.25	1.88	2.00	2.00	1.86	0.335	0.787
Cryptitis	2.88	2.00	2.50	2.25	2.25	0.315	0.088
Inflammatory infiltration of lamina propria	6.38	6.00	5.63	5.63	6.38	0.694	0.677
Inflammatory infiltration of epithelial cells	1.88	1.75	1.50	1.13	1.75	0.325	0.174
Bleeding	3.38^a^	2.00^b^	0.75^b^	1.00^b^	0.50^b^	0.694	<0.001
Total score	23.50^a^	17.63^a^	16.75^b^	14.88^b^	16.63^b^	2.250	0.006

1 Each mean represents 8 replicates.

2 CON, hens received basal diet; LD, hens received basal diet supplemented with 0.2 g/kg quercetin and 0.5 × 10^8^ CFU/kg *L. salivarius*; MD, hens received basal diet supplemented with 0.4 g/kg quercetin and 1 × 10^8^ CFU/kg *L. salivarius*; HD, hens received basal diet supplemented with 0.6 g/kg quercetin and 1.5 × 10^8^ CFU/kg *L. salivarius*; BM, hens received basal diet supplemented with 1.46 g/kg quercetin microcapsules (equivalent to 0.4 g/kg quercetin) and 1 × 10^8^ CFU/kg *L. salivarius*.

a-b Values within a row with no common superscripts differ significantly (*P* < 0.05).

#### The mRNA expression of tight junction proteins in the Ileal Mucosa

**Fig. 5:**
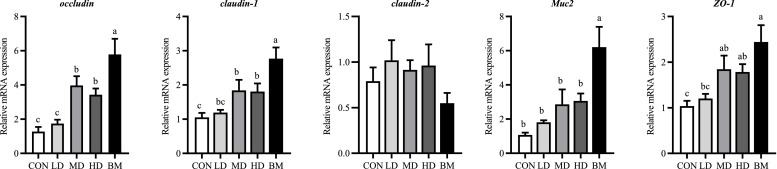
Effects of dietary quercetin and L. salivarius supplementation on the relative mRNA expression of tight junction proteins in ileum mucosa. CON, hens received basal diet; LD, hens received basal diet supplemented with 0.2 g/kg quercetin and 0.5 × 10^8^ CFU/kg *L. salivarius*; MD, hens received basal diet supplemented with 0.4 g/kg quercetin and 1 × 10^8^ CFU/kg *L. salivarius*; HD, hens received basal diet supplemented with 0.6 g/kg quercetin and 1.5 × 10^8^ CFU/kg *L. salivarius*; BM, hens received basal diet supplemented with 1.46 g/kg quercetin microcapsules (equivalent to 0.4 g/kg quercetin) and 1 × 10^8^ CFU/kg *L. salivarius*. ^a-c^ Means with different lowercase superscripts represent significant difference (*P* < 0.05).

As shown in Fig. 5, varying doses of quercetin and *L. salivarius* significantly enhanced the expression levels of *claudin-1* and *ZO-1* (*P* < 0.05). Compared to the CON group, the expression levels of *occludin, claudin-1*, and *ZO-1* were significantly elevated in the MD and HD groups (*P* < 0.05). Furthermore, in comparison to the CON group, the expression levels of *occludin, claudin-1, Muc2*, and *ZO-1* in the BM group were significantly increased (*P* < 0.01).

#### The mRNA Expression of Immune Response-related Genes in the Ileal Mucosa

**Fig. 6:**
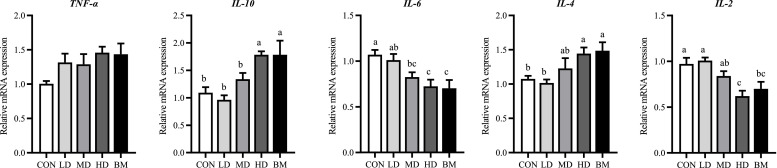
Effects of dietary quercetin and L. salivarius supplementation on the relative mRNA expression of immune response-related genes in ileum mucosa. CON, hens received basal diet; LD, hens received basal diet supplemented with 0.2 g/kg quercetin and 0.5 × 10^8^ CFU/kg *L. salivarius*; MD, hens received basal diet supplemented with 0.4 g/kg quercetin and 1 × 10^8^ CFU/kg *L. salivarius*; HD, hens received basal diet supplemented with 0.6 g/kg quercetin and 1.5 × 10^8^ CFU/kg *L. salivarius*; BM, hens received basal diet supplemented with 1.46 g/kg quercetin microcapsules (equivalent to 0.4 g/kg quercetin) and 1 × 10^8^ CFU/kg *L. salivarius*. ^a-c^ Means with different lowercase superscripts represent significant difference (*P* < 0.05).

As shown in Fig. 6, the expression levels of *IL-10* and *IL-4* in the HD and BM groups demonstrated a statistically significant increase compared to the CON group (*P* < 0.05). Conversely, the expression levels of *IL-6* and *IL-2* exhibited a statistically significant decrease (*P* < 0.05).

#### Ileal microbiota

**Fig. 7:**
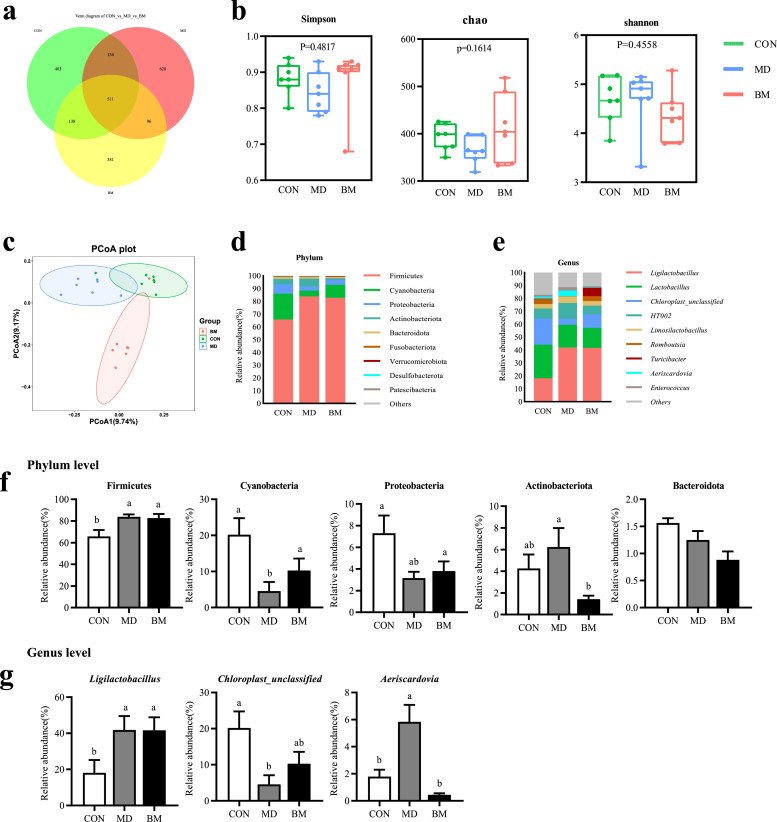
Dietary quercetin and *L. salivarius* intervention modulated the ileal microbial composition in laying hens. (a) Veen diagram of core amplicon sequence variants (ASVs) in the ileal digesta. (b) The α diversity parameters (Simpson, Chao and Shannon) of ileal microbiota. (c) Principal coordinate analysis (PCoA) of ileal microbiome in each group. Relative abundance of ileal microbial composition at the phylum (d) and genus (e) level. Relative abundance of ileal microbial community members at the phylum (f) and genus (g) level. CON, hens received basal diet; MD, hens received basal diet supplemented with 0.4 g/kg quercetin and 1 × 10^8^ CFU/kg *L. salivarius*; BM, hens received basal diet supplemented with 1.46 g/kg quercetin microcapsules (equivalent to 0.4 g/kg quercetin) and 1 × 10^8^ CFU/kg *L. salivarius*. ^a-c^ Means with different lowercase superscripts represent significant difference (*P* < 0.05).

Under the same dosage, quercetin microencapsulation demonstrates a more pronounced outcome. This may be attributed to the fact that microencapsulation enhances the concentration of quercetin in the posterior intestine, thereby exerting a greater influence on the microbial community. Building upon these findings, we subsequently characterized the responses of the ileal microbiota under both experimental conditions. After optimization, A total of 1,509,721 high-quality sequences with an average length of 412 bp were obtained from 21 samples, yielding an average of 71,891 sequences per sample and resulting in a total of 2,291 ASVs. Among these, there were 403 ASVs specific to the CON group, 628 ASVs specific to the MD group, and 381 ASVs specific to the BM group (Fig. 7a). The alpha diversity of the ileal microbiota in each group was analyzed based on ASVs data (Fig. 7b). The Simpson, Chao, and Shannon indices did not exhibit significant differences among the groups. However, a significant separation was observed in the Principal Coordinates Analysis (**PCoA**) based on the Jaccard index between the MD and BM groups (Fig. 7c), indicating that the MD and BM groups exhibited significant differences in the regulation of ileal microbiota.

The alterations in microbial abundance within the ileum of laying hens across the various groups were systematically analyzed. At the phylum level (Fig. 7d), the predominant phyla identified in all groups included Firmicutes, Cyanobacteria, and Proteobacteria. Notably, the relative abundance of Firmicutes in both the MD and BM groups was significantly higher than that in the CON group. At the genus level (Fig. 7e), the principal genera observed were *Ligilactobacillus, Lactobacillus, Chloroplast_unclassified*, and *HT002*.

Statistical analysis revealed a significant increase in the relative abundance of Firmicutes in both the MD and BM groups, as well as an increase in the relative abundance of Actinobacteriota in the MD group compared to the CON group (*P* < 0.05, Fig. 7f). Additionally, the relative abundance of Cyanobacteria and Proteobacteria in the MD group was significantly reduced compared to the CON group (*P* < 0.05, Fig. 7f). Furthermore, the analysis indicated a significant increase (*P* < 0.05) in the relative abundance of *Ligilactobacillus* in the MD and BM groups compared to the CON group (*P* < 0.05, Fig. 7g). In comparison to the CON group, the relative abundance of *chloroplast_unclassified* was significantly decreased in the MD group, while the abundance of Aeriscardovia was significantly increased (*P* < 0.05*,* Fig. 7g).

**Fig. 8:**
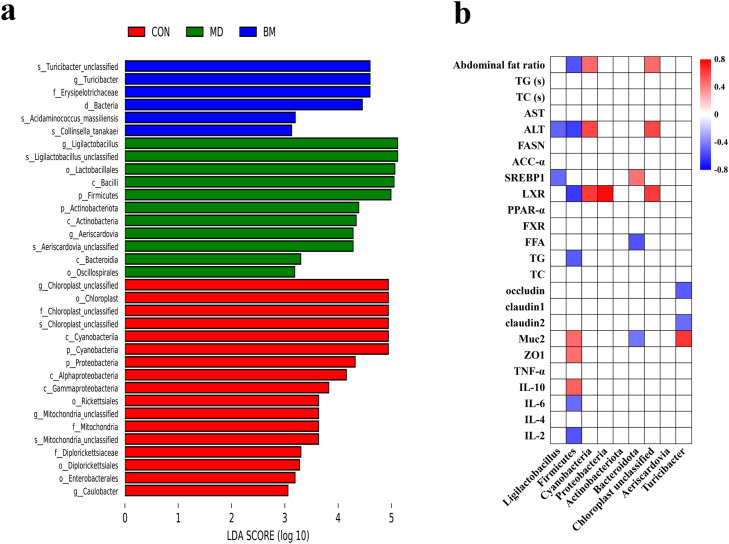
Dietary quercetin and *L. salivarius* intervention modulated the ileal microbial composition in laying hens. (a) linear discriminant analysis (LDA) combined effect size measurements (LEfSe) analysis of ileal microbiota. Person correlation between ileal microbiota and parameters. The intensity of the colors indicates the degree of the correlation, with red indicating significantly positive correlation and blue indicating negative correlation. CON, hens received basal diet; MD, hens received basal diet supplemented with 0.4 g/kg quercetin and 1 × 10^8^ CFU/kg *L. salivarius*; BM, hens received basal diet supplemented with 1.46 g/kg quercetin microcapsules (equivalent to 0.4 g/kg quercetin) and 1 × 10^8^ CFU/kg *L. salivarius*.

Abbreviations: *FXR*, farnesoid X receptor; *PPAR-α*, peroxisome proliferator activated receptor alpha; *SREBP1*, sterol-regulatory element-binding protein 1; *FASN*, fatty acid synthase; *LXR-α*, liver X receptors alpha; *ACC-α*, acetyl-CoA carboxylase alpha; FFA: Free fatty acids; HDL-C, high density lipoprotein cholesterol; LDL-C, low density lipoprotein cholesterol; TG, triglyceride; TC, total cholesterol; AST, aspartate aminotransferase; ALT, alanine aminotransferase.

Linear discriminant analysis Effect Size (**LEfSe**) was subsequently conducted to identify the microbial taxa that most effectively elucidate the differences among the three groups (*P* < 0.05, Fig. 8a). *Ligilactobacillus*, Lactobacillales, Bacilli, and Firmicutes were predominantly enriched in the MD group. Additionally, chloroplasts and cyanobacteria were found to be enriched in the BM group. Furthermore, *Turicibacter* and Erysipelotrichaceae were prevalent in the CON group. Pearson correlation analysis further confirmed that the modifications of ileal microbiota were associated with the combined application of quercetin and *L. salivarius* intervention. For instance, ALT, abdominal fat ratio, and LXR-α exhibited a positive correlation with Cyanobacteria and unclassified Chloroplasts, while showing a negative correlation with Ligilactobacillus and Firmicutes. Simultaneously, LXR-α was negatively correlated with Proteobacteria. *IL-6* and *IL-2* demonstrated a negative correlation with Firmicutes, whereas *IL-10* was positively correlated with Firmicutes. Hepatic TG were negatively correlated with Firmicutes, while hepatic FFA were negatively correlated with Bacteroidetes. *SREBP1, ZO-1, claudin-1*, and *Muc2* exhibited a positive correlation with Firmicutes and Turicibacter.

## Discussion

Recent studies have reported the positive effects of individual supplementation with quercetin and *L. salivarius* on the production performance and intestinal microbiota of laying hens (Xu et al., 2022; Liu et al., 2023a; Wei et al., 2024; Liu et al., 2024). However, the combined effects of these two components and their regulatory mechanisms on the production performance of laying hens have not been thoroughly investigated. In this study, the supplementation of quercetin in combination with *L. salivarius* significantly increased the laying rate, reduced the feed-to-egg ratio, and decreased abdominal fat. Moreover, this supplementation improved ileal barrier function and alleviated intestinal inflammatory reactions, and it was associated with alterations in the ileal microbiota. This study presents a novel strategy for the synergistic application of flavonoids and probiotics to enhance production performance in laying hens.

Microorganisms possess the capacity to biotransform the chemical structure of flavonoids (Paraiso et al., 2019; Meng et al., 2024), which may potentially hinder the combined application of probiotics and flavonoids. There is no apparent inhibition or consumption relationship between quercetin and *L. salivarius*, indicating that both can effectively reach their target organs and fulfill their respective functions.

Encapsulation technology has the potential to mitigate the chemical instability of quercetin, thereby enhancing its delivery to specific target sites for the effective execution of its biological functions (Wang et al., 2016; Caddeo et al., 2019; Zhang et al., 2024). This was confirmed by in vitro experiments, which revealed that encapsulation reduces the degradation of quercetin in the stomach and improves its bioavailability in the intestine.

In this study, the laying performance of laying hens with the combination of quercetin and *L. salivarius* was enhanced, manifested as an increase in laying rate and a decrease in ADFI and feed-to-egg ratio. Meanwhile, the optimal concentration of quercetin was determined to be 0.4 g/kg, as this level resulted in the maximum improvement in laying rate. This finding is consistent with results reported in previous studies (Liu et al., 2023a; Liu et al., 2014). Correspondingly, the results indicated that microencapsulated quercetin had the most significant effect on enhancing the laying ratio across all treatments. This improvement may be attributed to the enhanced sustained release and bioavailability of quercetin resulting from microencapsulation (Kandemir et al., 2022). These improvement result from the combined effects of quercetin and *L. salivarius*, which reduce the abdominal fat ratio and liver lipid deposition (Liu et al., 2023a; Liu et al., 2024). A high abdominal fat ratio indicates that a greater proportion of feed energy is allocated to fat deposition rather than egg production (Anene et al., 2023). Furthermore, a high abdominal fat ratio and liver lipid deposition are associated with an increased metabolic burden, which can impact hormone levels in laying hens, subsequently affecting both production performance and feed utilization (Chen et al., 2006). Our results suggest that the combined supplementation of quercetin and *L. salivarius* enhances the production performance of laying hens during the late laying period; however, the underlying mechanisms require further investigation.

The liver plays a pivotal role in lipid metabolism in laying hens, and maintaining a healthy state of hepatic lipid metabolism is crucial for ensuring optimal production performance (Zaefarian et al., 2019; Feng et al., 2023b). The liver is particularly susceptible to lipid deposition and oxidative stress during the late laying cycle (Li et al., 2015). Supplementation with quercetin and *L. salivarius* significantly reduced TG and TC levels in the liver, indicating a decrease in liver fat deposition and an improvement in liver health, which was further supported by the reduction in AST and ALT levels (Malnick et al., 2022). The reduction in serum TG levels was accompanied by a concomitant decrease in TC, HDL-C, and LDL-C levels, while a significant reduction in the abdominal fat ratio was also observed. Notably, microencapsulated quercetin demonstrated the most pronounced effects in ameliorating hepatic lipid deposition and liver injury, while reducing abdominal fat percentage. This may be attributed to the gradual release of microencapsulated quercetin in the intestine, which facilitates a more consistent cellular uptake and blood concentration, leading to a more stable and prolonged effect (Mandal et al., 2005; Vijayakumar et al., 2017). This suggests that microencapsulated quercetin may be a more potential form of application in laying hen production.

The results indicated that quercetin and *L. salivarius* suppressed the expression of lipogenesis genes, such as *ACC-α, SREBP-1*, and *LXR-α*, while enhancing the expression of *FXR*, a gene that inhibits lipogenesis. These regulatory effects may be related to the bile salt hydrolase activity of *L. salivarius*, which could influence the expression of key lipid metabolism genes in the ileum and liver, including *FXR, LXR-α*, and *PPAR-α* (Joyce et al., 2014a; Song et al., 2023). Bile salt hydrolases can directly regulate the composition of intestinal bile acids, while activation of the FXR can decrease lipid absorption in a bile acid-dependent manner (Clifford et al., 2021). Consequently, we observed a significant activation of FXR, which may mediate the observed reductions in triglycerides TG and TC. Similarly, quercetin has been shown to downregulate the expression of miR-122 in mouse liver, which indirectly leads to a decrease in *PPAR-α* mRNA levels and ultimately results in reduced liver lipid deposition (Gan et al., 2017). Additionally, it has been suggested that quercetin may regulate lipid metabolism gene expression by activating the mitogen-activated protein kinase pathway (Shen et al., 2023). Our findings suggest that the combination of quercetin and *L. salivarius* may have a synergistic effect on the expression of lipid metabolism genes; however, the underlying mechanisms warrant further investigation.

The maintenance of healthy intestinal morphology and barrier function, along with an optimal inflammatory state, forms the foundation of intestinal health (Jensen et al., 2023). Supplementation with quercetin and *L. salivarius* resulted in a higher ratio of villus height to crypt depth. This improvement can lead to an increase in the absorptive surface area of the gut, which may enhance the rate of nutrient absorption (Wilson et al., 2018). This is evidenced by the lower ADFI and feed-to-egg ratio observed in laying hens supplemented with quercetin and *L. salivarius*. A larger absorptive area is associated with a higher renewal rate of epithelial cells, potentially linked to the modulation of the intestinal inflammatory state by quercetin and *L. salivarius* (Dong et al., 2020; Liu et al., 2022b; Liu et al., 2024). An appropriate inflammatory state in the intestine may contribute to the maintenance of a healthy intestinal barrier function (Gadaleta et al., 2011; Feng et al., 2023c), as evidenced by increased mRNA expression levels of tight junction protein genes such as *occludin, claudin-1*, and *ZO-1*, along with alterations in the mRNA expression of intestinal inflammatory factor genes. The results indicate that dietary supplementation with quercetin and *L. salivarius* could enhance ileal barrier function and improve the intestinal inflammatory state, thereby promoting the maintenance of intestinal health and the production performance of laying hens.

Interestingly, the BM group (0.4 g/kg quercetin encapsulation) exhibited effects comparable to those of the HD group (0.6 g/kg quercetin), as evidenced by the regulation of lipid metabolism genes such as *ACC-α* and *FXR*, as well as genes associated with ileal inflammatory factors. Furthermore, the BM group demonstrated superior regulation of tight junction protein gene expression in the ileum. This mechanism may be attributed to the encapsulation process, which enhances both the quantity and duration of quercetin release in the ileum (Li et al., 2009; Caddeo et al., 2019; Hu et al., 2023).

Our previous results indicate that quercetin and *L. salivarius* lack interaction in vitro. The synergistic effect of quercetin and *L. salivarius* may be indirectly achieved through the interaction of intestinal microbiota (Khan et al., 2020; Liang et al., 2023). Encapsulated quercetin may exhibit enhanced quantity and duration in the ileum, potentially leading to distinct effects on the microbiota and mediating variations in regulatory effects (Lee et al., 2019; Ta et al., 2024). Therefore, we analyzed the impact of quercetin encapsulation on the ileal microbiota. The α-diversity of the microbial community was not significantly affected, which may be attributed to the resilience and resistance of the gut microbiota community (Fassarella et al., 2021). The relative abundance of Firmicutes increased, while Bacteroidetes decreased, resulting in an elevated Firmicutes/Bacteroidetes ratio. The abundance of Firmicutes has been shown to correlate positively with energy uptake (Koliada et al., 2017; Murugesan et al., 2018). Furthermore, it has been established that the Firmicutes/Bacteroidetes ratio plays a crucial role in indicating the status of gut bacteria, with an increase in this ratio being directly associated with improved production performance (Mariat et al., 2009; Xu et al., 2016).

The group supplemented with quercetin and *L. salivarius* showed an increase in the abundance of *Ligilactobacillus* and *Lactobacillus* spp., as well as *Aeriscardovia*. Lactobacillales are not only recognized as a significant class of probiotics but also as crucial commensal organisms within the chicken gut (Huang et al., 2018). These bacteria provide a wide range of benefits, including the inhibition of pathogen colonization and the enhancement of host intestinal health. Multiple species of Lactobacillales have been shown to regulate bile acid metabolism and lipid metabolism, thereby reducing serum cholesterol and TG levels (Joyce et al., 2014b; Jiang et al., 2023). Potential mechanisms of action may include the secretion of effector molecules, such as bile salt hydrolases, extracellular polysaccharides, and Short-chain fatty acids (**SCFA**), which regulate bile acid metabolism, lipid synthesis, and lipolysis (Horáčková et al., 2018; Nogal et al., 2021; Yilmaz et al., 2024). Furthermore, lactic acid produced by Lactobacillus fermentation can be utilized by SCFA producers through a cross-feeding model, resulting in beneficial effects of SCFA on nutrient digestibility and gut morphology (Moens et al., 2017). This potential model was also demonstrated by the enrichment of the MD group with Aeriscardovia, a genus belonging to the Bifidobacteriaceae family, which has been proposed as a key SCFA producer and has shown performance-enhancing effects on animal growth (Farooq and Wang, 2023). Additionally, Aeriscardovia has been shown to promote intestinal butyric acid production through cross-feeding (Lugli et al., 2017; Farooq and Wang, 2023; Zhao et al., 2024). In conclusion, the results indicate that dietary supplementation with quercetin and *L. salivarius* enhances the structure of the ileal microbiota in laying hens, which may elucidate the observed improvements in production performance and lipid metabolism.

In conclusion, our study demonstrated that the combination of quercetin and *L. salivarius* effectively reduced liver lipid deposition and the abdominal fat ratio, thus favoring enhancing the laying ratio and feed conversion efficiency. These findings provide novel insights into the combined application of quercetin and *L. salivarius* for enhancing the performance of laying hens.

## Declaration of competing interest

The authors have declared that they have no conflicts of interest.

## References

[bib0001] Anene D.O., Akter Y., Groves P.J., Horadagoda N., Liu S.Y., Moss A., Hutchison C., O'Shea C.J. (2023). Association of feed efficiency with organ characteristics and fatty liver haemorrhagic syndrome in laying hens. Sci. Rep..

[bib0003] Bijle M.N., Neelakantan P., Ekambaram M., Lo E.C.M., Yiu C.K.Y. (2020). Effect of a novel synbiotic on Streptococcus mutans. Sci. Rep..

[bib0004] BNO Team (2024). https://biologynotesonline.com/spread-plate-method-principle/.

[bib0005] Caddeo C., Gabriele M., Fernàndez-Busquets X., Valenti D., Fadda A.M., Pucci L., Manconi M. (2019). Antioxidant activity of quercetin in eudragit-coated liposomes for intestinal delivery. Int. J. Pharm..

[bib0007] Chen S.E., McMurtry J.P., Walzem R.L. (2006). Overfeeding-induced ovarian dysfunction in broiler breeder hens is associated with lipotoxicity. Poult. Sci..

[bib0008] Choi E.J., Bae S.M., Ahn W.S. (2008). Antiproliferative effects of quercetin through cell cycle arrest and apoptosis in human breast cancer MDA-MB-453 cells. Arch. Pharm. Res..

[bib0009] Clifford B.L., Sedgeman L.R., Williams K.J., Morand P., Cheng A., Jarrett K.E., Chan A.P., Brearley-Sholto M.C., Wahlström A., Ashby J.W., Barshop W., Wohlschlegel J., Calkin A.C., Liu Y., Thorell A., Meikle P.J., Drew B.G., Mack J.J., Marschall H.U., Tarling E.J., de Aguiar Vallim T.Q. (2021). FXR activation protects against NAFLD via bile-acid-dependent reductions in lipid absorption. Cell Metab..

[bib0012] Dong Y.Y., Lei J.Q., Zhang B.K. (2020). Effects of dietary quercetin on the antioxidative status and cecal microbiota in broiler chickens fed with oxidized oil. Poult. Sci..

[bib0013] Dos Santos A.S., T. M. de Albuquerque R., de Brito Alves J.L., de Souza E.L (2019). Effects of quercetin and resveratrol on in vitro properties related to the functionality of potentially probiotic *Lactobacillus* strains. Front. Microbiol..

[bib0014] Eid H.M., Nachar A., Thong F., Sweeney G., Haddad P.S. (2015). The molecular basis of the antidiabetic action of quercetin in cultured skeletal muscle cells and hepatocytes. Pharmacogn. Mag..

[bib0015] Farooq M.Z., Wang X.K., Yan X.H. (2023). Effects of *Aeriscardovia aeriphila* on growth performance, antioxidant functions, immune responses, and gut microbiota in broiler chickens. J. Zhejiang. Univ. Sci. B.

[bib0016] Fassarella M., Blaak E.E., Penders J., Nauta A., Smidt H., Zoetendal E.G. (2021). Gut microbiome stability and resilience: elucidating the response to perturbations in order to modulate gut health. Gut.

[bib0017] Fathi M.M., Galal A., Ali U.M., Abou-Emera O.K. (2019). Physical and mechanical properties of eggshell as affected by chicken breed and flock age. Br. Poult. Sci..

[bib0018] Feng J., Ma H., Yue Y.R., Wang L.J., Hao K.Y., Zhang Y.N., Li J.H., Xiang Y.J., Min Y.N. (2023). Saikosaponin a ameliorates diet-induced fatty liver via regulating intestinal microbiota and bile acid profile in laying hens. Poult. Sci..

[bib0019] Feng J., Li Z.R., Ma H., Yue Y.R., Hao K.Y., Li J.H., Xiang Y.J., Min Y.N. (2023). Quercetin alleviates intestinal inflammation and improves intestinal functions via modulating gut microbiota composition in LPS-challenged laying hens. Poult. Sci..

[bib0020] Feng Y.Q., Zhang M.H., Liu Y., Yang X.Y., Wei F.X., Jin X.L., Liu D., Guo Y.M., Hu Y.F. (2023). Quantitative microbiome profiling reveals the developmental trajectory of the chicken gut microbiota and its connection to host metabolism. Imeta.

[bib0021] Gadaleta R.M., van Erpecum K.J., Oldenburg B., Willemsen E.C., Renooij W., Murzilli S., Klomp L.W., Siersema P.D., Schipper M.E., Danese S., Penna G., Laverny G., Adorini L., Moschetta A., van Mil S.W. (2011). Farnesoid X receptor activation inhibits inflammation and preserves the intestinal barrier in inflammatory bowel disease. Gut.

[bib0022] Gadalla H.H., El-Gibaly I., Soliman G.M., Mohamed F.A., El-Sayed A.M. (2016). Amidated pectin/sodium carboxymethylcellulose microspheres as a new carrier for colonic drug targeting: development and optimization by factorial design. Carbohydr. Polym..

[bib0023] Gan C.C., Ni T.W., Yu Y., Qin N., Chen Y., Jin M.N., Duan H.Q. (2017). Flavonoid derivative (Fla-CN) inhibited adipocyte differentiation via activating AMPK and up-regulating microRNA-27 in 3T3-L1 cells. Eur. J. Pharmacol..

[bib0024] Gu Y.F., Chen Y.P., Jin R., Wang C., Wen C., Zhou Y.M. (2021). Age-related changes in liver metabolism and antioxidant capacity of laying hens. Poult. Sci..

[bib0025] Horáčková Š., Plocková M., Demnerová K. (2018). Importance of microbial defence systems to bile salts and mechanisms of serum cholesterol reduction. Biotechnol. Adv..

[bib0026] Hu J.L., Jiao W.C., Tang Z.Y., Wang C.Q., Li Q., Wei M., Song S.Y., Du L.N., Jin Y.G. (2023). Quercetin inclusion complex gels ameliorate radiation-induced brain injury by regulating gut microbiota. Biomed. PharmacOther.

[bib0027] Huang P., Zhang Y., Xiao K.P., Jiang F., Wang H.C., Tang D.Z., Liu D., Liu B., Liu Y.S., He X., Liu H., Liu X.B., Qing Z.X., Liu C.H., Huang J.L., Ren Y.W., Yun L., Yin L.J., Lin Q., Zeng C., Su X.G., Yuan J.Y., Lin L., Hu N.X., Cao H.L., Huang S.W., Guo Y.M., Fan W., Zeng J.G. (2018). The chicken gut metagenome and the modulatory effects of plant-derived benzylisoquinoline alkaloids. Microbiome.

[bib0028] Jensen B.A.H., Heyndrickx M., Jonkers D., Mackie A., Millet S., Naghibi M., Pærregaard S.I., Pot B., Saulnier D., Sina C., Sterkman L.G.W., Van den Abbeele P., Venlet N.V., Zoetendal E.G., Ouwehand A.C. (2023). Small intestine vs. colon ecology and physiology: why it matters in probiotic administration. Cell Rep. Med..

[bib0029] Jiang M.W., Li F.Y., Liu Y.H., Gu Z.L., Zhang L.H., Lee J., He L.Q., Vatsalya V., Zhang H.G., Deng Z.B., Zhang X., Chen S.Y., Guo G.L., Barve S., McClain C.J., Feng W.K. (2023). Probiotic-derived nanoparticles inhibit ALD through intestinal miR194 suppression and subsequent FXR activation. Hepatology.

[bib0030] Joyce S.A., Shanahan F., Hill C., Gahan C.G. (2014). Bacterial bile salt hydrolase in host metabolism: potential for influencing gastrointestinal microbe-host crosstalk. Gut. Microbes..

[bib0031] Joyce S.A., MacSharry J., Casey P.G., Kinsella M., Murphy E.F., Shanahan F., Hill C., Gahan C.G. (2014). Regulation of host weight gain and lipid metabolism by bacterial bile acid modification in the gut. PNAS.

[bib0032] Kandemir K., Tomas M., McClements D.J., Capanoglu E. (2022). Recent advances on the improvement of quercetin bioavailability. Trends Food Sci. Tech.

[bib0033] Khan S., Moore R.J., Stanley D., Chousalkar K.K. (2020). The gut microbiota of laying hens and its manipulation with prebiotics and probiotics to enhance gut health and food safety. Appl. Environ. Microbiol..

[bib0034] Kim E.S., Kim D.Y., Lee J.S., Lee H.G. (2021). Quercetin delivery characteristics of chitosan nanoparticles prepared with different molecular weight polyanion cross-linkers. Carbohydr. Polym..

[bib0035] Koliada A., Syzenko G., Moseiko V., Budovska L., Puchkov K., Perederiy V., Gavalko Y., Dorofeyev A., Romanenko M., Tkach S., Sineok L., Lushchak O., Vaiserman A. (2017). Association between body mass index and Firmicutes/bacteroidetes ratio in an adult Ukrainian population. BMC. Microbiol..

[bib0036] Lee J.Y., Han G.G., Kim E.B., Choi Y.J. (2017). Comparative genomics of *Lactobacillus salivarius* strains focusing on their host adaptation. Microbiol. Res..

[bib0037] Lee S., Kirkland R., Grunewald Z.I., Sun Q., Wicker L., de La Serre C.B. (2019). Beneficial effects of non-encapsulated or encapsulated probiotic supplementation on microbiota composition, intestinal barrier functions, inflammatory profiles, and glucose tolerance in high fat fed rats. Nutrients..

[bib0038] Li H., Wang T., Xu C., Wang D., Ren J., Li Y., Tian Y., Wang Y., Jiao Y., Kang X., Liu X. (2015). Transcriptome profile of liver at different physiological stages reveals potential mode for lipid metabolism in laying hens. BMC. Genomics..

[bib0039] Li H., Zhao X., Ma Y., Zhai G., Li L., Lou H. (2009). Enhancement of gastrointestinal absorption of quercetin by solid lipid nanoparticles. J. Control Release.

[bib0040] Liang X.X., Fu Y.W., Niu K.M., Zhai Z.Y., Shi H.X., Wang R., Yin Y.L. (2023). Dietary *Eucommia ulmoides* leaf extract improves production performance by altering serum metabolic profiles and gut bacteria in aged laying hens. Anim. Nutr..

[bib0041] Liu H.N., Liu Y., Hu L., Suo L., Zhang L., Jin F., Feng X.A., Teng N., Li Y. (2014). Effects of dietary supplementation of quercetin on performance, egg quality, cecal microflora populations, and antioxidant status in laying hens. Poult. Sci..

[bib0042] Liu J.Y., Fu Y.X., Zhou S.S., Zhao P.Y., Zhao J., Yang Q.L., Wu H., Ding M.Y., Li Y. (2023). Comparison of the effect of quercetin and daidzein on production performance, anti-oxidation, hormones, and cecal microflora in laying hens during the late laying period. Poult. Sci..

[bib0043] Liu L., Zhou Z., Hong Y., Jiang K., Yu L., Xie X., Mi Y., Zhu S.J., Zhang C., Li J. (2022). Transplantion of predominant lactobacilli from native hens to commercial hens could indirectly regulate their ISC activity by improving intestinal microbiota. Microb. Biotechnol..

[bib0044] Liu S.Y., Loo Y.T., Li Z.Z., Ng K. (2023). Alginate-inulin-chitosan based microspheres alter metabolic fate of encapsulated quercetin, promote short chain fatty acid production, and modulate pig gut microbiota. Food Chem..

[bib0045] Liu W., Liu J., Li D.P., Han H.X., Yan H.X., Sun Y., Lei Q.X., Wang J., Zhou Y., Cao D.G., Li H.M., Li F.W. (2024). Effect of *Lactobacillus salivarius* SNK-6 on egg quality, intestinal morphology, and cecal microbial community of laying hens. Poult. Sci..

[bib0046] Liu Y.L., Yan T., Ren Z.Z., Yang X.J. (2021). Age-associated changes in caecal microbiome and their apparent correlations with growth performances of layer pullets. Anim. Nutr..

[bib0047] Liu Y., Li L., Yan H., Ning Z., Wang Z. (2022). *Lactobacillus salivarius* SNK-6 activates intestinal mucosal immune system by regulating cecal microbial community structure in laying hens. Microorganisms..

[bib0048] Lou J., Duan H.L., Qin Q., Teng Z.P., Gan F.X., Zhou X.F., Zhou X. (2023). Advances in oral drug delivery systems: challenges and opportunities. Pharmaceutics..

[bib0049] Lugli G.A., Milani C., Turroni F., Duranti S., Mancabelli L., Mangifesta M., Ferrario C., Modesto M., Mattarelli P., Jiří K., Sinderen D.V., Ventura M. (2017). Comparative genomic and phylogenomic analyses of the *Bifidobacteriaceae* family. BMC. Genomics..

[bib0050] Malnick S.D.H., Alin P., Somin M., Neuman M.G. (2022). Fatty liver disease-alcoholic and non-alcoholic: similar but different. Int. J. Mol. Sci..

[bib0051] Mandal A.K., Das N. (2005). Sugar coated liposomal flavonoid: a unique formulation in combating carbontetrachloride induced hepatic oxidative damage. J. Drug Target..

[bib0052] Mariat D., Firmesse O., Levenez F., Guimarăes V., Sokol H., Doré J., Corthier G., Furet J.-P. (2009). The firmicutes/bacteroidetes ratio of the human microbiota changes with age. BMC. Microbiol..

[bib0053] Meng X., Xia C.L., Wu H.C., Gu Q., Li P. (2024). Metabolism of quercitrin in the colon and its beneficial regulatory effects on gut microbiota. J. Sci. Food Agric..

[bib0054] Moens F., Verce M., Vuyst L.D. (2017). Lactate-and acetate-based cross-feeding interactions between selected strains of lactobacilli, bifidobacteria and colon bacteria in the presence of inulin-type fructans. Int. J. Food Microbiol..

[bib0055] Mukhopadhyay P., Sarkar K., Bhattacharya S., Bhattacharyya A., Mishra R., Kundu P.P. (2014). pH sensitive N-succinyl chitosan grafted polyacrylamide hydrogel for oral insulin delivery. Carbohydr. Polym..

[bib0056] Murugesan S., Nirmalkar k., Hoyo-Vadillo C., García-Espitia M., Ramírez-Sánchez D., García-Mena J. (2018). Gut microbiome production of short-chain fatty acids and obesity in children. Eur. J. Clin. Microbiol. Infect. Dis..

[bib0057] Nair M.P., Mahajan S., Reynolds J.L., Aalinkeel R., Nair H., Schwartz S.A., Kandaswami C. (2006). The flavonoid quercetin inhibits proinflammatory cytokine (tumor necrosis factor alpha) gene expression in normal peripheral blood mononuclear cells via modulation of the NF-kappa beta system. Clin. Vaccine Immunol..

[bib0058] Nogal A., Valdes A.M., Menni C. (2021). The role of short-chain fatty acids in the interplay between gut microbiota and diet in cardio-metabolic health. Gut. Microbes..

[bib86] NRC (1994). Nutrient Requirements of Poultry.

[bib0059] Obianwuna U.E., Qiu K., Wang J., Zhang H.J., Qi G.H., Huang L.L., Wu S.G. (2023). Effects of dietary *Clostridium butyricum* and fructooligosaccharides, alone or in combination, on performance, egg quality, amino acid digestibility, jejunal morphology, immune function, and antioxidant capacity of laying hens. Front. Microbiol..

[bib0060] Paraiso I.L., Plagmann L.S., Yang L.P., Zielke R., Gombart A.F., Maier C.S., Sikora A.E., Blakemore P.R., Stevens J.F. (2019). Reductive metabolism of xanthohumol and 8-prenylnaringenin by the intestinal bacterium *eubacterium ramulus*. Mol. Nutr. Food Res..

[bib0062] Schmucker S., Hofmann T., Sommerfeld V., Huber K., Rodehutscord M., Stefanski V. (2021). Immune parameters in two different laying hen strains during five production periods. Poult. Sci..

[bib0063] Shen M., Li T., Feng Y., Wu P., Serrano B.R., Barcenas A.R., Qu L., Zhao W.G. (2023). Effects of quercetin on granulosa cells from prehierarchical follicles by modulating MAPK signaling pathway in chicken. Poult. Sci..

[bib0064] Shini A., Shini S., Bryden W.L. (2019). Fatty liver haemorrhagic syndrome occurrence in laying hens: impact of production system. Avian Pathol..

[bib0065] Simonetti G., Buiarelli F., Bernardini F., Filippo P.D., Riccardi C., Pomata D. (2022). Profile of free and conjugated quercetin content in different Italian wines. Food Chem..

[bib0066] Song Z.W., Feng S., Zhou X.C., Song Z.X., Li J., Li P. (2023). Taxonomic identification of bile salt hydrolase-encoding lactobacilli: modulation of the enterohepatic bile acid profile. Imeta.

[bib0067] Ta L.P., Corrigan S., Tselepis C., Iqbal T.H., Ludwig C., Horniblow R.H. (2024). Gastrointestinal-inert prebiotic micro-composites improve the growth and community diversity of mucosal-associated bacteria. J. Control Release.

[bib0068] Vijayakumar A., Baskaran R., Jang Y.S., Oh S.H., Yoo B.K. (2017). Quercetin-loaded solid lipid nanoparticle dispersion with improved physicochemical properties and cellular uptake. AAPS. PharmSciTech..

[bib0069] Wang F., Zou P., Xu S., Wang Q., Zhou Y., Li X., Tang L., Wang B., Jin Q., Yu D., Li W. (2022). Dietary supplementation of Macleaya cordata extract and Bacillus in combination improve laying performance by regulating reproductive hormones, intestinal microbiota and barrier function of laying hens. J. Anim. Sci. Biotechnol..

[bib0070] Wang W.Y., Sun C.X., Mao L., Ma P.H., Liu F.G., Yang J., Gao Y.X. (2016). The biological activities, chemical stability, metabolism and delivery systems of quercetin: a review. Trends Food. Sci. Tech.

[bib0071] Wang Y.P., Jin T.H., Zhang N.B., Li J.K., Wang Y., Kulyar M.F.-E.-A., Han Z.Q., Li Y.Z. (2021). Effect of stocking density and age on physiological performance and dynamic gut bacterial and fungal communities in Langya hens. Microb. Cell Fact..

[bib0072] Wei Y., Liu Y.F., Li G., Guo Y.M., Zhang B.K. (2024). Effects of quercetin and genistein on egg quality, lipid profiles, and immunity in laying hens. J. Sci. Food Agric..

[bib0073] Wilson F.D., Cummings T.S., Barbosa T.M., Williams C.J., Gerard P.D., Peebles E.D. (2018). Comparison of two methods for determination of intestinal villus to crypt ratios and documentation of early age-associated ratio changes in broiler chickens. Poult. Sci..

[bib0074] Xiao S.S., Mi J.D., Mei L., Liang J., Feng K.X., Wu Y.B., Liao X.D., Wang Y. (2021). Microbial diversity and community variation in the intestines of layer chickens. Animals. (Basel).

[bib0075] Xu C., Wei F.X., Yang X.Y., Feng Y.Q., Liu D., Hu Y.F. (2022). *Lactobacillus salivarius* CML352 isolated from Chinese local breed chicken modulates the gut microbiota and improves intestinal health and egg quality in late-phase laying hens. Microorganisms..

[bib0076] Xu Y.H., Yang H.X., Zhang L.L., Su Y.H., Shi D.H., Xiao H.D., Tian Y.M. (2016). High-throughput sequencing technology to reveal the composition and function of cecal microbiota in Dagu chicken. BMC. Microbiol..

[bib0077] Yilmaz B., Arslan N., Şahin T.Ö., Ağadündüz D., Ozogul F., Rocha J.M.F. (2024). Unveiling the impact of lactic acid bacteria on blood lipid regulation for cardiovascular health. Fermentation.

[bib0078] Zaefarian F., Abdollahi M.R., Cowieson A., Ravindran V. (2019). Avian liver: the forgotten organ. Animals. (Basel).

[bib0079] Zhang F.J., Liu J.G., Uyanga V.A., Tang C.Y., Qu Y.N., Qin X., Chen Y.L., Liu Y.Q. (2024). Preparation and functional properties of rice bran globulin-chitooligosaccharide-quercetin-resveratrol covalent complex. J. Sci. Food Agric..

[bib0080] Zhao S.N., Lau R.M., Zhong Y., Chen M.H. (2024). Lactate cross-feeding between *bifidobacterium* species and *megasphaera indica* contributes to butyrate formation in the human colonic environment. Appl. Environ. Microbiol..

[bib0082] Zhu L., Liao R., Huang J., Xiao C., Yang Y., Wang H., He D., Yan H., Yang C. (2022). *Lactobacillus salivarius* SNK-6 regulates liver lipid metabolism partly via the miR-130a-5p/MBOAT2 pathway in a NAFLD model of laying hens. Cells.

[bib0083] Zhu X.Q., Dai X.J., Zhao L.J., Li J., Zhu Y.H., He W.J., Guan X.L., Wu T., Liu L., Song H.P., Lei L. (2024). Quercetin activates energy expenditure to combat metabolic syndrome through modulating gut microbiota-bile acids crosstalk in mice. Gut. Microbes..

[bib0084] Zhuang M.M., Zhang X., Cai J. (2024). Microbiota-gut-brain axis: interplay between microbiota, barrier function and lymphatic system. Gut. Microbes..

